# Construction of an Effector–Target Interaction Network for Identification of Immune‐Related Effectors in *Ralstonia pseudosolanacearum*

**DOI:** 10.1111/mpp.70280

**Published:** 2026-06-09

**Authors:** Bingbing Xue, Yongxiao Xie, Yang Zhang, Wenyan Zhong, Zhuoyuan Tan, Jing Lv, Yongjun Lin, Lifang Ruan

**Affiliations:** ^1^ Hainan Research Institute of Huazhong Agricultural University Sanya China; ^2^ State Key Laboratory of Agricultural Microbiology College of Life Science and Technology, Huazhong Agricultural University Wuhan China; ^3^ National Key Laboratory of Crop Genetic Improvement and National Center of Plant Gene Research, College of Life Science and Technology Huazhong Agricultural University Wuhan China

## Abstract

Bacterial wilt caused by *Ralstonia pseudosolanacearum* is a devastating soil‐borne disease that results in significant losses of various crops worldwide. The type III secretion system (T3SS) secretes a suite of effectors into host cells, which is a major driver of disease development. These effectors do not act in isolation but form an interconnected network called the effectorome, functioning collectively through extensive crosstalk. Constructing large‐scale effector–target interaction networks via high‐throughput protein interaction assays is one of the primary approaches to studying effector function at the effectorome level. However, false positives inherent in high‐throughput protein interaction methods hinder precise understanding of effector function. Here, we performed Gene Ontology (GO) enrichment analysis on a previously reported large‐scale *Ralstonia pseudosolanacearum* effector– *Arabidopsis thaliana* target interaction network and found that effectors primarily interact with immune response‐related proteins. Subsequently, we validated the interactions between immune‐related target proteins and their corresponding effectors using one‐to‐one yeast two‐hybrid assays, generating a validated immune‐related effector‐target subnetwork. Finally, analyses of reactive oxygen species (ROS) burst, callose deposition, and immune‐related gene transcription revealed that the effectors in the subnetwork exhibit immune‐suppressive functions, demonstrating the potential of the effector–target network in understanding the pathogenic mechanism at the effectorome level.

## Graphical abstract

Based on the previously reported effector–target interaction network, we refined an immune‐related subnetwork via one‐to‐one yeast two‐hybrid assays in *Ralstonia pseudosolanacearum*, and showed all its effectors exhibit immune‐suppressive functions, demonstrating the power of refined effector–target networks to dissect effectoromes function.

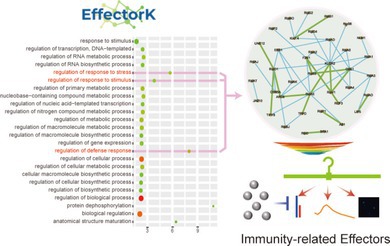

To counteract threats from various pathogens, plants have evolved a sophisticated immune defence system that detects pathogen invasion and eliminates or restricts infections to maintain health and growth (Zhou and Zhang 2020). One of the most critical virulence weapons employed by pathogenic bacteria is the type III secretion system, which injects a repertoire of effector proteins into host cells (Landry et al. 2020). Effectors do not function in isolation but exhibit extensive interactions. Multiple effector knockout analyses in *Ralstonia pseudosolanacearum* (Lei et al. 2020), *Xanthomonas oryzae* (Yan et al. 2023) and *Pseudomonas syringae* (Cunnac et al. 2011) have shown that individual or a few effector knockouts often do not cause a significant reduction in pathogenicity, indicating widespread redundant interactions among effectors. Moreover, certain effectors can suppress effector‐triggered immunity (ETI) responses induced by other effectors, which represents another classic form of effector interplay (Wei et al. 2018; Martel et al. 2022). For example, *R. pseudosolanacearum* RipAC regulates the phosphorylation of SGT1, thereby affecting NLR protein homeostasis and suppressing the hypersensitive response (HR) induced by effectors such as RipAA (Yu et al. 2020; Nakano et al. 2021). *P. syringae* AvrPtoB promotes the degradation of ADR1‐L1, a key component of ETI signalling, via the ubiquitin–proteasome pathway, thereby inhibiting ETI responses induced by effectors like HopAD1 (Wang, Chen, et al. 2023; Wei et al. 2015). A systematic study on the landscape of ETI induction by effectoromes revealed that up to 96.8% of *P. syringae* strains contain at least one effector homologue capable of inducing ETI in *Arabidopsis thaliana* Col‐0 (Laflamme et al. 2020). Given that most strains in this study are considered pathogenic, this suggests that ETI‐suppressing effectors may be widespread (Martel et al. 2022). The extensive interplay among effectors has promoted a “holistic” perspective for understanding effector function, with the entire set of effectors in a pathogenic species referred to as an effectorome, effectomes or effector network (Bundalovic‐Torma et al. 2022; Sanchez‐Garrido et al. 2022; Arroyo‐Velez et al. 2020). For clarity and alignment with our recent work, we use effectorome here to refer specifically to multiple such repertoires.

Research on effectoromes is still in its early stages, and several approaches have been proposed to decipher their functions and mechanisms. Multiple effector knockout, which involves sequentially knocking out effectors in a given pathogen strain in a random order, is a powerful strategy for studying functional redundancy within effectoromes. This approach identified the “minimal effectorome” essential for basic pathogenicity in *P. syringae* (Cunnac et al. 2011). Multiple effector knockout to generate effectorome‐deficient *P. syringae* mutants (Δ36E), followed by individual or combined expression of effectors and analysis of their virulence or ETI‐inducing functions, can elucidate cooperative interactions among effectors (Wei et al. 2018; Martel et al. 2022). Ruiz‐Bedoya et al. (2023) demonstrated that expressing individual effectors in *P. syringae* DC3000 Δ36E, which were termed “effector clones”, did not confer virulence. However, when 35 of these effector clones were combined into a mixed population, termed a “metaclone,” full pathogenicity was restored. This indicates that effectors can act as cooperative public goods (Ruiz‐Bedoya et al. 2023). The metaclone system allows flexible assembly of effector combinations and holds promise for investigating effector–effector interactions and functional groupings.

High‐throughput protein–protein interaction screening to systematically identify host targets of effectors and construct effector–host target interaction networks is an effective method for deciphering effectorome functions. For instance, high‐throughput yeast two‐hybrid (Y2H) assays have been used to systematically screen for targets of effectors from *P. syringae* , *Hyaloperonospora arabidopsidis* and *Golovinomyces orontiii*, leading to the construction of a plant–pathogen protein–protein interactome network (Mukhtar et al. 2011; Weßling et al. 2014). Studies have shown that effectors from the same or different pathogens converge on a common set of key plant proteins, suggesting potential redundant or synergistic interactions among these effectors. Host targets of effectors from *R. pseudosolanacearum* and *X. campestris* have been identified using yeast two‐hybrid systems (González‐Fuente et al. 2020). By integrating these large‐scale cross‐species plant–pathogen interactome networks and extensive literature, a comprehensive online knowledge‐sharing platform called EffectorK has been constructed, providing robust support for systematic analysis of effectorome functions (González‐Fuente et al. 2020).

*Ralstonia pseudosolanacearum* is a devastating soil‐borne pathogenic bacterium widely distributed in tropical, subtropical and some temperate regions worldwide. It infects over 250 plant species, including important economic crops such as tomato, potato and tobacco, and is ranked as the second most significant bacterial pathogen (Mansfield et al. 2012; Jiang et al. 2017; Genin and Denny 2012). González‐Fuente et al. (2020) identified target proteins of *R. pseudosolanacearum* effectors in *A. thaliana* using high‐throughput Y2H assays at the effectorome level. Functional analysis of these target proteins provided insights into the roles of the *R. pseudosolanacearum* effectorome. We analysed all these target proteins (involving 186 target proteins) by Gene Ontology (GO) enrichment and found that significant enrichment of GO terms including “protein dephosphorylation”, “regulation of defence response”, “anatomical structure maturation”, “regulation of response to stress” and “regulation of response to stimulus” (Figure 1A). Notably, “regulation of defence response”, “regulation of response to stress” and “regulation of response to stimulus” are all immune defence‐related GO terms. These three GO terms are hierarchical (Figure S1).

**FIGURE 1:**
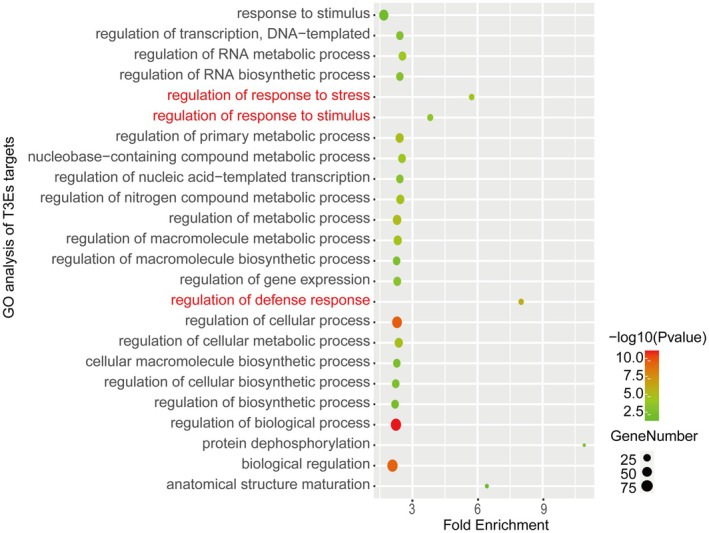
GO enrichment analysis of *Arabidopsis thaliana* targets of *Ralstonia pseudosolanacearum* effectors. The position of bubbles represents fold enrichment, the colour of bubbles represents −log_10_(*p* value), and the size of bubbles represents the number of genes corresponding to each function. GO terms related to immunity (the focus of this study) are marked in red font.

High‐throughput Y2H assays are a powerful tool for identifying effector targets at the effectorome level and analysing their functional characteristics. However, the inherent false‐positive rate of high‐throughput Y2H assays limits the accuracy of effector function evaluation. This study focused on the 16 target genes corresponding to the GO term “regulation of defence response”, 23 effectors corresponding to these target genes, and 54 pairwise interactions formed between them; these components were subsequently used to construct an immune‐related subnetwork (Figure 2A and Table S2). The effectors involved were predicted to have immune regulation functions, but this inference was subject to false positives from Y2H. To eliminate these artefacts, one‐by‐one Y2H assays were performed to validate the high‐throughput screening results, yielding a small but more reliable immune‐related subnetwork (Figures 2A and S2). Figure 2A, nodes represent effectors or host targets, and edges (lines) represent interactions between effectors and host targets. Thin blue lines denote 54 interactions identified in high‐throughput screening, while thick green lines represent 16 interactions validated by one‐by‐one Y2H. The network had a core consisting of effectors (e.g., RipV1, RipAE) as hub nodes and their host targets (e.g., APC8, OBE1, KLCR2). We observed increased mRNA abundance of *A. thaliana* immune‐related genes, such as *APC8* and *KLR2* (the reverse transcription‐quantitative PCR [RT‐qPCR] primer sequences are listed in Table S1), during infection with the *R. pseudosolanacearum* T3SS‐deficient mutant (Δ*hrpY*). In contrast, their expression levels were relatively decreased upon wild‐type infection, suggesting that specific type III effectors suppress the transcription of these genes (Figure S3C).

**FIGURE 2:**
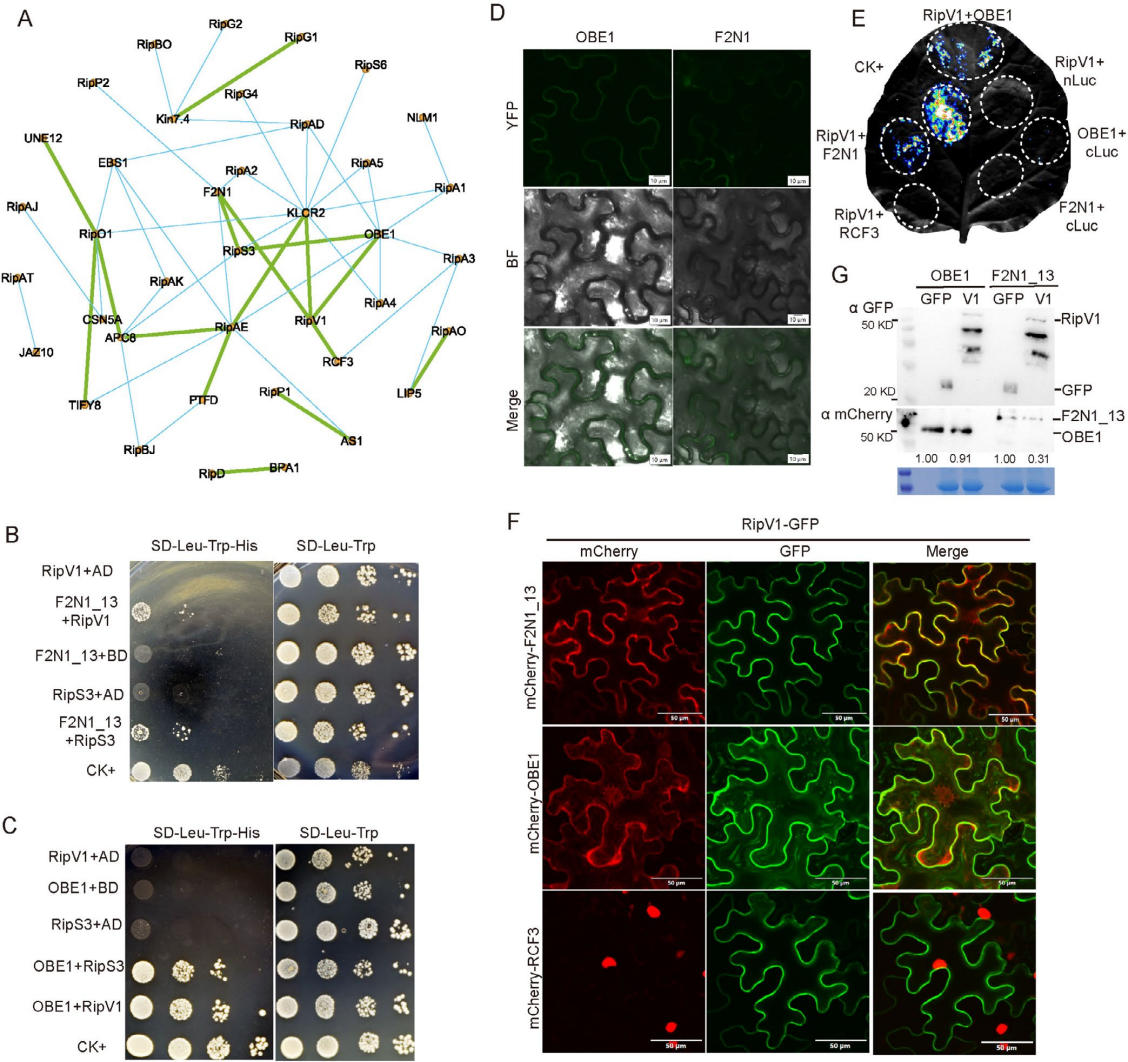
Construction and validation of the effector–target interaction network related to immunity. (A) The interaction network of *Arabidopsis thaliana* target proteins assigned to the GO term “regulation of defence response” and their corresponding effectors of *Ralstonia pseudosolanacearum* was constructed. Orange–yellow nodes represent target proteins or effectors. Edges between nodes represent interactions, where thin blue lines indicate positive interactions identified by high‐throughput yeast two‐hybrid (Y2H) screening, and thick green lines indicate positive interactions verified by one‐by‐one yeast two‐hybrid assay. (B) Validation of the interaction between RipV1, RipS3, and F2N1_13 by Y2H. Colonies from left to right show the growth of designated strains after 10‐fold serial dilution. SD−Leu/Trp/His was used to select the interaction, and SD−Leu/Trp was used as the control. (C) Validation of the interaction between RipV1, RipS3, and OBE1 by Y2H. (D) Bimolecular fluorescence complementation (BiFC) assay was used to verify that RipV1 can interact with OBE1 and F2N1_13. (E) Luciferase complementation assay (LCA) was used to verify that RipV1 can interact with OBE1 and F2N1_13. (F) Fluorescence colocalisation analysis of RipV1 and its potential targets. The target proteins F2N1_13, OBE1, and RCF3 were N‐terminally tagged with mCherry, and RipV1 was C‐terminally tagged with GFP. These constructs were co‐expressed pairwise in *Nicotiana benthamiana*, and fluorescence was visualised by confocal microscopy. (G) Western blot analysis of the effect of RipV1 on the protein homeostasis of OBE1 and F2N1_13. After co‐expression of GFP or RipV1‐GFP with host targets mCherry‐OBE1and mCherry‐F2N1_13, the accumulation levels of each protein were analysed using anti‐GFP or anti‐mCherry antibodies. The protein accumulation levels of OBE1or F2N1_13 were quantified using ImageJ.

Pathogenic bacterial effectors often target multiple host proteins and interfere with diverse physiological processes, exhibiting functional versatility. RipV1 interacted with F2N1_13 and OBE1 (Figure 2B–E), further demonstrating that functional versatility is a common property of effectors. This versatility promotes extensive connections between effectors, ultimately forming a functional ensemble of the effectorome. For example, RipAE and RipV1 jointly interacted with KLCR2, RipS3 and RipV1 jointly interacted with OBE1, and RipO1 interacted with RipAE and APC8 (Figure 2A).

Given that Y2H may introduce false positives due to forced nuclear localisation of proteins, this study further validated RipV1 interactions with OBE1, RCF3 and F2N1_13 using bimolecular fluorescence complementation (BiFC) and luciferase complementation assays (LCA) (details of experimental procedures are provided in File S1). We found that only OBE1 and F2N1_13 interacted with RipV1, suggesting a potential false‐positive interaction between RipV1 and RCF3 (Figures 2D,E and S3A,B). Subsequently, RipV1 was fused with a GFP tag, while OBE1, RCF3, and F2N1_13 were fused with mCherry tags. After co‐expressing these constructs in *Nicotiana benthamiana* leaves, their subcellular localisations were observed. F2N1_13 was primarily localised to the plasma membrane and cytoplasm, whereas OBE1 exhibited multiple localisation in the cytoplasm, plasma membrane and nucleus and showed clear colocalisation with RipV1. In contrast, RCF3 was exclusively localised to the nucleus and did not colocalise with RipV1 (Figure 2F). RipV1 has been demonstrated to possess ubiquitin ligase activity (Choi et al. 2026), potentially mediating the degradation of its targets. Western blot analysis revealed that RipV1 slightly promoted the degradation of OBE1 but significantly enhanced the degradation of F2N1_13 (Figure 2G). However, whether this degradation occurs through the ubiquitin–proteasome pathway remains to be experimentally determined. These results indicate that OBE1 and F2N1_13 are functional targets of RipV1.

A common principle in effector biology is that if a host target of an effector has immune regulation functions, the effector itself probably possesses immune regulation activity. In the immune‐related target–effector interaction network constructed in this study, the involved effectors (RipO1, RipAE, RipV1, RipS3, RipAO, RipG1 and RipP1) were predicted to have immune regulation functions. These effectors were fused to mCherry tags and transiently expressed in *N. benthamiana* by *Agrobacterium tumefaciens* infiltration. Their subcellular localisation was observed using confocal laser scanning microscopy. As shown in Figure 3A, the intracellular fluorescent signals of RipAE, RipG1, RipO1, RipV1 and RipAO were stronger than the surrounding background regions, with signals along the cell outline significantly enhanced, indicating their potential localisation to the plasma membrane and cytoplasm. Among these, RipAE, RipG1, RipO1 and RipAO also exhibited localisation to the nucleus or its vicinity, suggesting they might also localise to the nucleus or endoplasmic reticulum structures. RipS3 localised to the nucleus. RipP1 was excluded from further analysis due to severe cell death induced by its transient expression in *N. benthamiana*. All tested effectors were readily expressed in the *N. benthamiana* transient system and were thus suitable for subsequent immune‐related functional characterisation.

**FIGURE 3:**
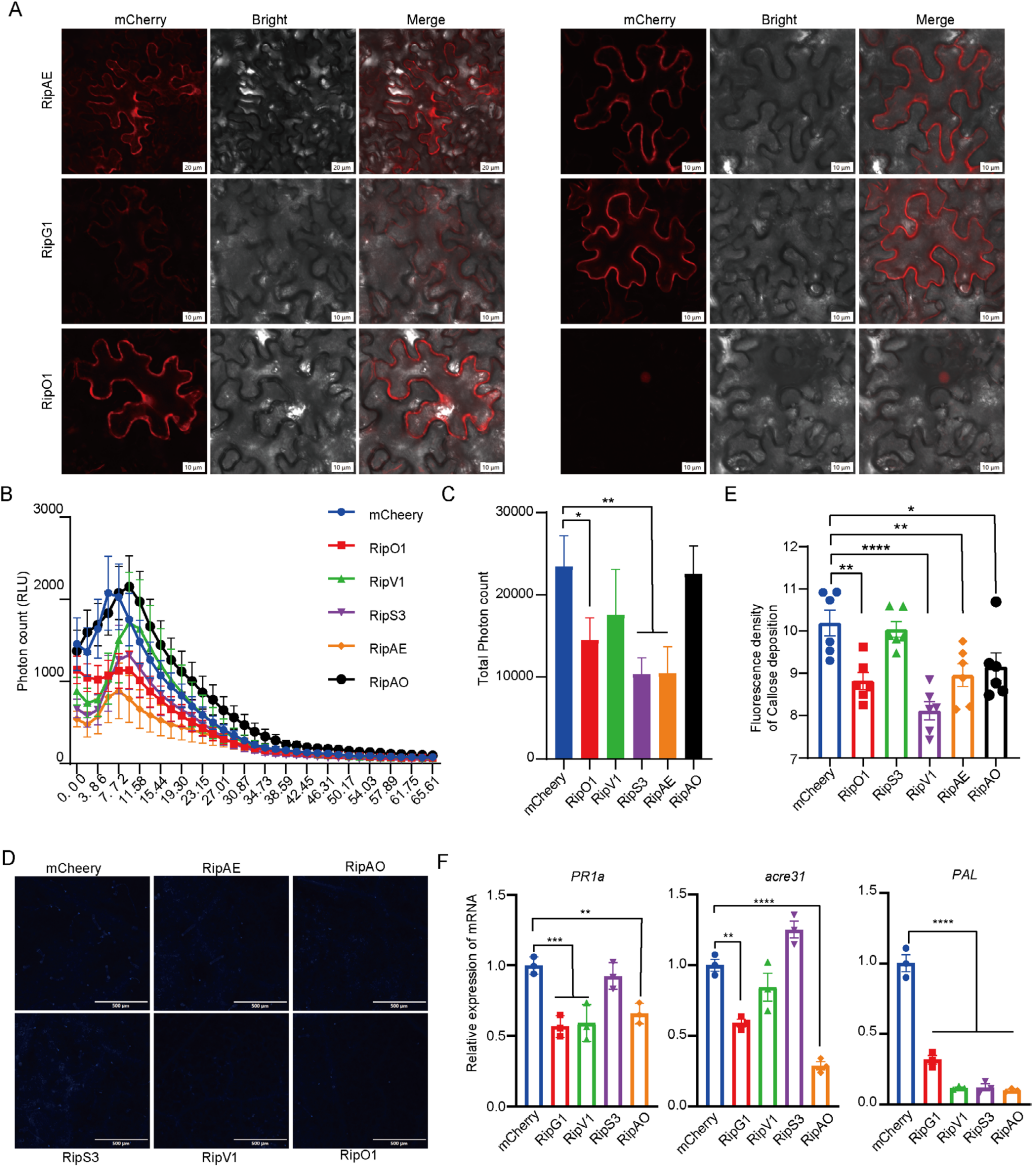
Preliminary characterisation of immune‐related effectors of *Ralstonia pseudosolanacearum*. (A) Subcellular localisation analysis of immune‐related effectors of *R. pseudosolanacearum*. The N‐terminus of each effector was fused to an mCherry tag, and subcellular localisation was analysed via *Agrobacterium*‐mediated transient expression in *Nicotiana benthamiana* combined with confocal fluorescence microscopy. (B) flg22‐triggered reactive oxygen species (ROS) burst in *N. benthamiana* leaves transiently expressing the effector. Effectors were transiently expressed in *N. benthamiana* leaves, and ROS responses were assessed after flg22 treatment. A higher photon count (relative luminescence units, RLU) indicates a stronger ROS burst intensity. Data are presented as mean ± SEM of 16 technical replicates. (C) Total accumulated photon counts of the flg22‐induced ROS burst in (B). (D) Quantitative assessment of flg22‐induced callose deposition in *N. benthamiana* leaves transiently expressing effectors. Callose deposition was visualised by aniline blue staining followed by fluorescence microscopy imaging. (E) Quantitative analysis of flg22‐induced callose deposition in (D). Data are presented as mean ± SEM of 6 technical replicates. (F) Reverse transcription‐quantitative real‐time PCR analysis of *PR1a*, *Acre31* and *PAL* mRNA expression in *N. benthamiana* leaves transiently expressing effectors. Data represent mean ± SEM (*n* = 3 biological replicates) normalised to *ACTIN2* transcript levels (*p < 0.05; **p < 0.01; ***p < 0.001, ****p < 0.0001).

Subsequently, the effect of each effector on flg22‐triggered reactive oxygen species (ROS) burst was measured using luminol chemiluminescence. As shown in Figure 3B,C, RipAE, RipS3 and RipO1 significantly suppressed the ROS burst, demonstrating their immune suppression function. RipV1 showed a weak and unstable inhibitory effect on ROS burst (Figure 3B). The effect of each effector on flg22‐induced callose deposition was then measured using aniline blue fluorescent staining. As shown in Figure 3D,E, RipAE, RipAO, RipO1 and RipV1 significantly inhibited callose deposition. Subsequently, RT‐qPCR analysis was performed to assess the effect of each effector on the mRNA transcriptional activation of the flg22‐induced immune‐related gene, inclued *PR1a*, *acre31* and *PAL*. As shown in Figure 3D, at 12 h post‐flg22 treatment, RipG1 and RipAO significantly suppressed the transcriptional activation of *PR1a*, *acre31* and *PAL*; RipV1 suppressed the transcriptional activation of *acre31* and *PAL*; while RipS3 only suppressed the expression of *PAL*. These results collectively indicate that effectors targeting immune‐related host components exhibit immune regulation functions.

Building upon the effector–target interaction network constructed by high‐throughput protein–protein interaction analysis (González‐Fuente et al. 2020), this study focused on immune‐related functions to construct a subnetwork and validate the interactions involving effector–target pairs. It is important to highlight that González‐Fuente et al. pioneered the de novo construction of an effector–target interaction network, performing large‐scale screening of effector interactors against an *Arabidopsis* cDNA library.; however, due to inherent limitations of high‐throughput methods such as relaxed control settings and potential nonspecific interactions in the library, their network contained relatively high false positives. Our study focused on immune‐related proteins and their corresponding effectors, validating interactions through one‐to‐one Y2H assays that theoretically yield lower false positives, a conclusion supported by BiFC and LCA of representative interactions. Moreover, we further used the effector–target network to preliminarily infer effector functions by reverse deduction from target proteins and validate these functions. Therefore, while the network by González‐Fuente et al. prioritised comprehensiveness and breadth, our study narrows to an immune‐related subnetwork, emphasising credibility and predictability of effector function. Analysis of the immune‐related subnetwork further demonstrated functional characteristics of effectors: (1) Effectors exhibit versatility by interacting with multiple distinct host targets (Bundalovic‐Torma et al. 2022). For instance, RipV1 interacts with both OBE1 and F2N1_13. (2) Effectors often converge on functionally critical targets (Mukhtar et al. 2011); for example, KLCR2, OBE1 and F2N1_13 interact with multiple distinct effectors. (3) The versatility and convergence of effectors lead to extensive functional connections between effectors, thereby forming an integrated effector network.

Although the interactions in the constructed immune‐related subnetwork were relatively sparse, and some effectors were isolated outside the network, we hypothesise that this is due to false negatives from the Y2H assay itself. For example, a considerable number of effectors or targets in this study exhibited autoactivation or cytotoxicity in the Y2H system, preventing subsequent interaction analysis (data not shown). Moreover, the classic Y2H system exhibits significantly reduced efficiency in detecting protein–protein interactions when one or both interactors contain transmembrane domains, which can also contribute to false negatives (Brückner et al. 2009). Supporting this hypothesis, it has been confirmed that RipD interacts with BPA1 and participates in immune regulation (Xue et al. 2025); however, BPA1 is not the only target of RipD. Another study demonstrated that RipD can also interact with vesicle‐associated membrane proteins (VAMPs) to suppress immunity (Wang, Yu, et al. 2023), suggesting that although only one target effector was identified in this study, other uncharacterised targets may still exist. Nevertheless, there may also be other immune regulatory effectors and their targets that are not covered by the immune‐related subnetwork constructed in this study.

The research methods for effectoromes are limited, primarily because effector functional characterisation is often a low‐throughput process that is time‐consuming and labour‐intensive (Arroyo‐Velez et al. 2020). The improved‐confidence effector–target interaction network established in this study includes a series of effectors with distinct immune regulatory functions. For example, RipAO does not affect the flg22‐induced ROS burst but significantly inhibits callose deposition and the transcription of immune‐related genes; RipV1 weakly inhibits the ROS burst but significantly suppresses the transcription of immune‐related genes. These results suggest that large‐scale, credible effector–target interaction networks are effective tools for high‐throughput functional characterisation of effectors.

## Author Contributions

**Wenyan Zhong:** data curation, investigation. **Lifang Ruan:** conceptualization, funding acquisition, writing – review and editing, writing – original draft, project administration, resources, supervision, visualization, methodology. **Yang Zhang:** data curation, investigation. **Yongxiao Xie:** data curation, investigation, validation, formal analysis, visualization. **Yongjun Lin:** methodology, supervision, resources, writing – review and editing, project administration. **Bingbing Xue:** methodology, conceptualization, investigation, visualization, funding acquisition, writing – original draft, writing – review and editing, formal analysis. **Jing Lv:** data curation, investigation. **Zhuoyuan Tan:** data curation, investigation.

## Funding

This work was supported by National Key Research and Development Program of China, (Grant 2024YFD1200203), Fundamental research funds for the central university of Huazhong Agricultural University (Grant 2662024SKPY001), Major Special Project for the Development of Agricultural Microbial Industry in Hubei Province (Grant NYWSWZX2025‐2027‐08) and the Hubei Provincial Natural Science Foundation of China (Grant JCZRQN202400263).

## Conflicts of Interest

The authors declare no conflicts of interest.

## Supporting information


**Figure S1:** Venn diagram of target genes included in immune‐related GO terms. The GO terms “regulation of defence response” is a key process targeted by effectors.

**Figure S2:** One‐to‐one yeast two‐hybrid assay validating effector‐immune‐related target interactions. In a panel, the left side represents SD medium deficient in Leu and Trp (serving as a control to show yeast growth viability), and the right side represents SD medium deficient in Leu/Trp/His (used for screening effector‐target pairs with protein interactions). From top to bottom, a 10‐fold serial dilution of yeast cell suspension is shown. The interaction of BPA1 and ACD11 was used as positive control (CK+).

**Figure S3:** Negative control for bimolecular fluorescence complementation analysis of RipV1 and its interacting proteins. (A,B) F2N1‐nYFP, RCF3‐nYFP, and OBE1‐nYFP were co‐expressed with cYFP; RipV1 was co‐expressed with nYFP. Corresponding images were captured by confocal microscopy. (C) Reverse transcription‐quantittaive PCR analysis of the effects of GMI1000 effectoromes on the transcriptional expression of key immune‐related components in the network. Using a needle‐free syringe, GMI1000, GMI1000 (Δ*hrpY*), or 10 mM MgCl₂ was infiltrated into *Arabidopsis thaliana* Col‐0 leaves; RNA was extracted 12 h post‐infiltration, and the accumulation levels of mRNA for key effector targets were quantified.

**File S1:** Experimental procedures.

**Table S1:** Oligonucleotides and primers used in this study.

**Table S2:** Details of components in the immune‐related effector‐target network.

## Data Availability

Data sharing not applicable to this article as no datasets were generated or analysed during the current study.
