# Supplementary material for: Construction of an Effector–Target Interaction Network for Identification of Immune‐Related Effectors in Ralstonia pseudosolanacearum
Source: Mol Plant Pathol. 2026 Jun 9;27(6):e70280. doi: 10.1111/mpp.70280 (PMC13250401; doi:10.1111/mpp.70280)
Supplement: Supplementary file 1 — Figure S1: Venn diagram of target genes included in immune‐related GO terms. The GO terms “regulation of defence response” is a key process targeted by effectors. [file MPP-27-e70280-s002.docx]

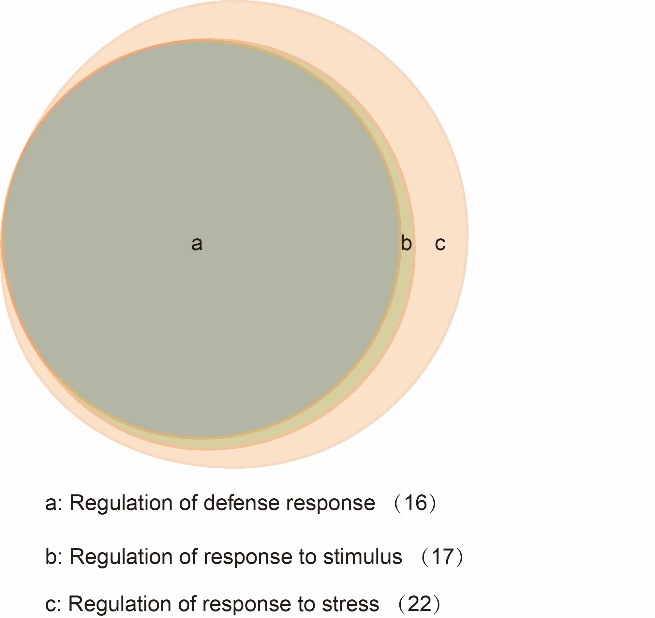


Fig S1 Venn diagram of target genes included in immune - related GO terms. The GO terms “regulation of defense response” is a key process targeted by effectors.
