# Supplementary material for: Construction of an Effector–Target Interaction Network for Identification of Immune‐Related Effectors in Ralstonia pseudosolanacearum
Source: Mol Plant Pathol. 2026 Jun 9;27(6):e70280. doi: 10.1111/mpp.70280 (PMC13250401; doi:10.1111/mpp.70280)
Supplement: Supplementary file 2 — Figure S2: One‐to‐one yeast two‐hybrid assay validating effector‐immune‐related target interactions. In a panel, the left side represents SD medium deficient in Leu and Trp (serving as a control to show yeast growth viability), and the right side represents SD medium deficient in Leu/Trp/His (used for screening effector‐target pairs with protein interactions). From top to bottom, a 10‐fold serial dilution of yeast cell suspension is shown. The interaction of BPA1 and ACD11 was used as positive control (CK+). [file MPP-27-e70280-s004.docx]

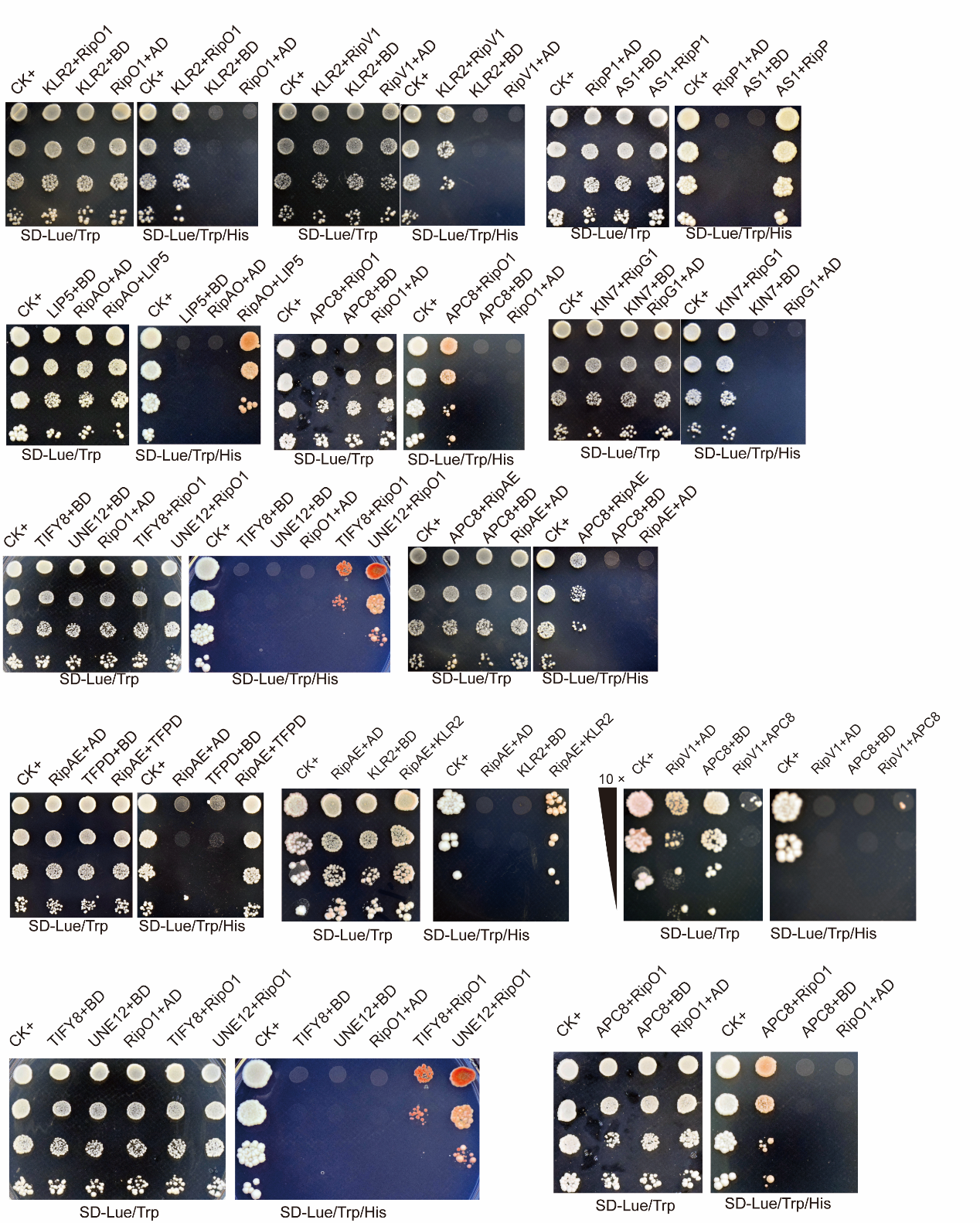


Figure S2：One-to-one yeast two-hybrid assay validating effector-immune-related target interactions. In a panel, the left side represents SD medium deficient in Leu and Trp (serving as a control to show yeast growth viability), and the right side represents SD medium deficient in Leu/Trp/His (used for screening effector-target pairs with protein interactions). From top to bottom, a 10-fold serial dilution of yeast cell suspension is shown. The interaction of BPA1 and ACD11 was used as positive control (CK+).
