# Supplementary material for: Construction of an Effector–Target Interaction Network for Identification of Immune‐Related Effectors in Ralstonia pseudosolanacearum
Source: Mol Plant Pathol. 2026 Jun 9;27(6):e70280. doi: 10.1111/mpp.70280 (PMC13250401; doi:10.1111/mpp.70280)
Supplement: Supplementary file 3 — Figure S3: Negative control for bimolecular fluorescence complementation analysis of RipV1 and its interacting proteins. (A,B) F2N1‐nYFP, RCF3‐nYFP, and OBE1‐nYFP were co‐expressed with cYFP; RipV1 was co‐expressed with nYFP. Corresponding images were captured by confocal microscopy. (C) Reverse transcription‐quantittaive PCR analysis of the effects of GMI1000 effectoromes on the transcriptional expression of key immune‐related components in the network. Using a needle‐free syringe, GMI1000, GMI1000 (ΔhrpY), or 10 mM MgCl₂ was infiltrated into Arabidopsis thaliana Col‐0 leaves; RNA was extracted 12 h post‐infiltration, and the accumulation levels of mRNA for key effector targets were quantified. [file MPP-27-e70280-s003.docx]

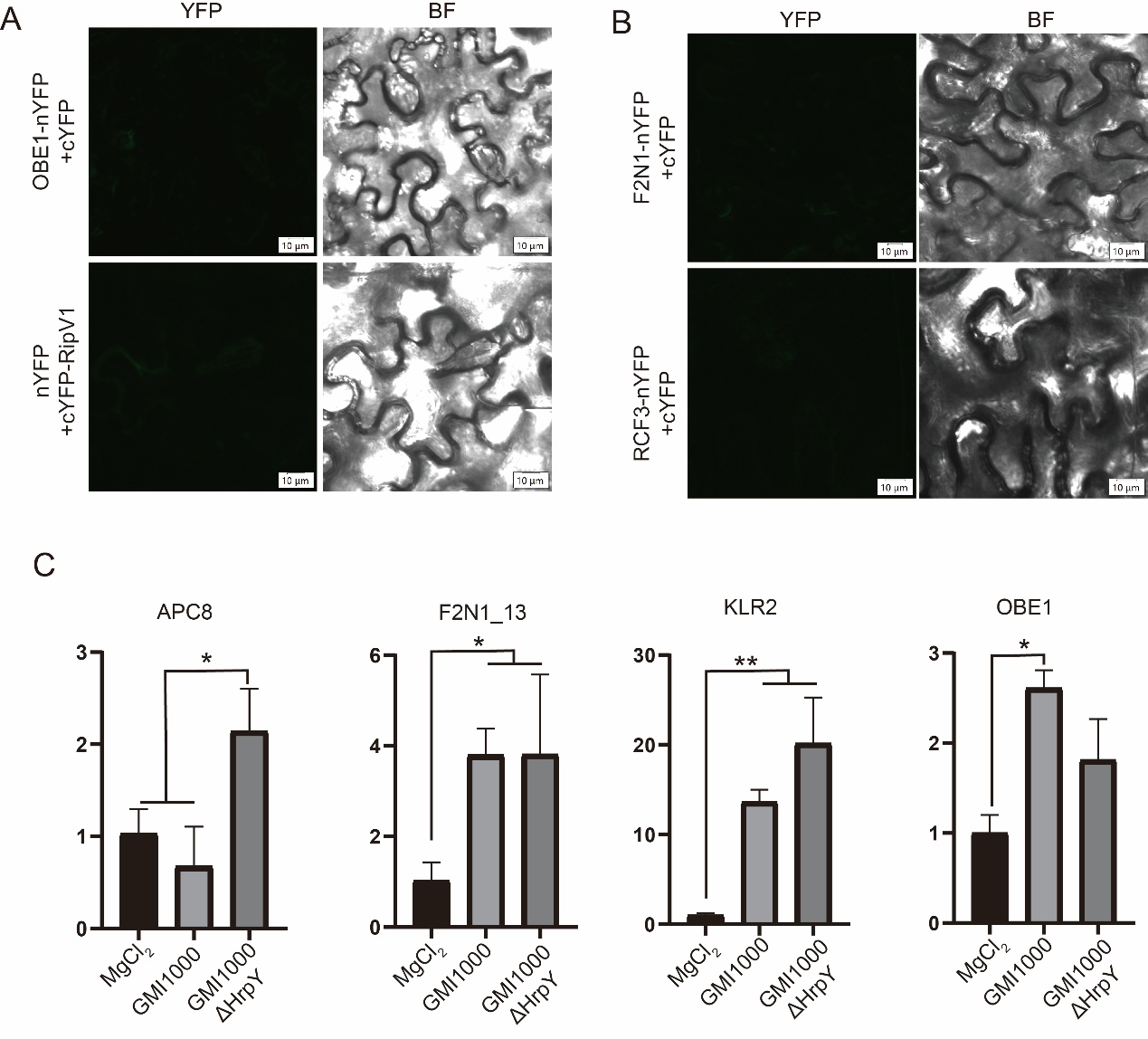


Figure S3: Negative control for BiFC analysis of RipV1 and its interacting proteins.

(A-B) F2N1-nYFP, RCF3-nYFP, and OBE1-nYFP were co-expressed with cYFP; RipV1 was co-expressed with nYFP. Corresponding images were captured by confocal microscopy.

(C) Quantitative real-time PCR (qRT-PCR) analysis of the effects of GMI1000 effectoromes on the transcriptional expression of key immune-related components in the network. Using a needle-free syringe, GMI1000, Δ*hpY* GMI1000, or 10 mM MgCl₂ was infiltrated into *A. thaliana* Col-0 leaves; RNA was extracted 12 h post-infiltration, and the accumulation levels of mRNA for key effector targets were quantified.
