# Supplementary material for: Construction of an Effector–Target Interaction Network for Identification of Immune‐Related Effectors in Ralstonia pseudosolanacearum
Source: Mol Plant Pathol. 2026 Jun 9;27(6):e70280. doi: 10.1111/mpp.70280 (PMC13250401; doi:10.1111/mpp.70280)
Supplement: Supplementary file 4 — File S1: Experimental procedures. [file MPP-27-e70280-s005.docx]

**Experimental Procedures**

**Plants, Bacterial Strains and Growth Conditions**

*R. pseudosolanacearum* GMI1000 was cultured in BG medium (10 g/L bactopeptone, 1 g/L casamino acids, 1 g/L yeast extract, and 5 g/L glucose) at 28°C, and genomic DNA was extracted for DNA amplification and cloning. *Saccharomyces cerevisiae* Y187 and AH109 were cultured in YPDA medium (10.0 g/L Yeast extract, 20.0 g/L Peptone, 20.0 g/L glucose, and 0.03 g/L Adenine sulfate) or SD medium (6.7 g/L yeast nitrogen base without amino acids, 20 g/L glucose and 100 mL 10×Dropout solution) at 28°C. *Agrobacterium tumefaciens* GV3101 and *Escherichia coli* DH5α was cultured in LB medium (10g/L Tryptone, 5g/L Yeast extract, 10g/L NaCl).

*Nicotiana benthamiana* and *Arabidopsis thaliana were* grown in a standard greenhouse under conditions of 22–25°C, 16-h light/8-h dark photoperiod, 60–70% relative humidity, and 120–150 μmol/m²/s light intensity.

**GO enrichment analysis and visualization**

The interaction network between *R. pseudosolanacearum* effectors and *A. thaliana* candidate targets was downloaded from the EffectorK database(González-Fuente et al., 2020). The *A. thaliana* candidate targets were subjected to GO enrichment analysis using the PANTHER Classification System. Finally, the results were visualized using the ggPlot package in R studio, with the following main parameters: ggplot(data, aes (x = Fold Enrichment, y=Biological Process))+geom_point(aes(size=Gene Number, color = -log10 (Pvalue))) + scale_colour_gradient (low = "green", high = "red") + theme (text = element_text(size = 12)).

**Yeast Two-Hybrid Assay**

Effector genes from *R. pseudosolanacearum* were amplified using primers listed in Table S1. The PCR products were purified and digested with NdeI and EcoRI (TaKaRa Biotechnology Co., Ltd), then mixed with the linearized pGBKT7 vector (digested with the same enzymes). For *Arabidopsis* target genes, PCR amplification and purification were performed, followed by digestion with EcoRI and XhoI (TaKaRa Biotechnology Co., Ltd), and ligation with the linearized pGADT7 vector. The ligation products were then transformed into *Escherichia coli*.

The recombinant pGBKT7 plasmids were transformed into *Saccharomyces cerevisiae* strain AH109, while the recombinant pGADT7 plasmids were transformed into strain Y187. AH109 and Y187 strains containing the target effector-prey pairs were cultured overnight in YPDA medium at 30°C. The cultures were centrifuged to collect the cells, which were resuspended in 2× YPDA medium. The cell suspension was spotted onto 2× YPDA plates, followed by mating at 30°C for 48 h.

After mating, the diploid yeast cells were resuspended in SD medium and spread onto SD/-Leu/-Trp medium to select for successful mating (this medium lacks leucine and tryptophan, which are supplemented by the pGADT7 and pGBKT7 vectors, respectively). Positive colonies from the SD/-Leu/-Trp medium were picked and cultured at 30°C with shaking (200 rpm) for 48 h. The cultures were then serially diluted (10-fold) and spotted onto SD/-Leu/-Trp medium and SD/-Leu/-Trp/-His medium. Colonies that grew on both media were considered positive for effector-target interactions.

**Agrobacterium-Mediated Transient Expression, Bimolecular Fluorescence Complementation (BiFC), Luciferase Complementation Assay (LCA) and Subcellular Localization Analysis**

Effector genes from *R. pseudosolanacearum* were amplified using primers listed in Table S1. The PCR products were purified, then mixed with the linearized pCambia1300S (mCherry) vector (digested with the same enzymes). Equal volumes of 2× clone mix were added, mixed thoroughly, and incubated at 50°C for 30 min. The products were transformed into *E. coli*. After recombinant plasmid construction, the plasmids were transformed into *A. tumefaciens* GV3101 via electroporation. The *A. tumefaciens* were activated and cultured overnight in LB medium at 28°C. The cells were collected by centrifugation, resuspended in induction buffer (0.2 mM acetosyringone, 10 mM MgCl₂, 10 mM MES) to an OD₆₀₀ of 0.6. Four-week-old *N. benthamiana* plants were infiltrated with the bacterial suspension using a syringe (without a needle) from the abaxial side of the leaves, allowing the suspension to infiltrate the mesophyll tissue.

For BiFC analysis, the effector gene was cloned into the pZY101-cYFP vector (with cYFP fused to the C-terminus of the effector), and the target gene was cloned into the pZY101-nYFP vector (with nYFP fused to the N-terminus of the target protein). The recombinant plasmids were transformed into *A. tumefaciens* strains. The *A. tumefaciens* cells carrying the effector and target constructs were mixed in a 1:1 ratio and infiltrated into *N. benthamiana* leaves as described above.Fluorescence microscopy was performed using a confocal laser scanning microscope (FV1000, Olympus) with a 20× air immersion objective (numerical aperture = 0.8) at 2–3 days post-infiltration. The subcellular localization of the effector and the interaction between the effector and target protein were observed by detecting YFP fluorescence (excitation: 514 nm; emission: 527 nm) and mCherry fluorescence (excitation: 587 nm; emission: 610 nm).

For the luciferase complementation assay (LCA) analysis, effector genes were fused to the C- terminal fragment of luciferase (cLuc), while host targets were fused to the N- terminal fragment of luciferase (nLuc). The successfully constructed recombinant vectors were separately transformed into *A. tumefaciens* strain GV3101. The *Agrobacterium* suspensions containing the effector-target pairs to be tested were mixed and infiltrated into leaves of *N. benthamiana*, followed by incubation in a greenhouse for approximately 60 h. A cotton swab was used to apply the substrate solution (1 mM D-luciferin, 0.1% Triton X-100) onto the abaxial surface of the *N. benthamiana* leaves. After incubation in the dark for approximately 10 min, images were captured using a plant molecular imaging system (Lumazone Pylon 2048B, Princeton, USA).

**Plant Total Protein Extraction and Western Blot**Leaves of *N. benthamiana* transiently expressing the target protein were sampled, weighed, and ground into a fine powder in liquid nitrogen. To 0.1 g of the dry powder, 0.4 mL of total protein extraction buffer (4.5 M urea, 80 µM SDS, 20 µM Tris-HCl (pH 6.8), and 20 µM β-mercaptoethanol) was added. After thorough mixing, the mixture was incubated on ice for 5 min. Subsequently, the sample was centrifuged at 12,000 × g for 15 min at 4°C, and the supernatant was collected and mixed with 5× SDS sample buffer. The resulting sample was boiled in a water bath for 10 min, then centrifuged again at 12,000 × g for 10 min at 4°C, and the supernatant was taken as the protein sample.

The protein sample was separated by 12% SDS-PAGE and transferred to a nitrocellulose membrane. The membrane was then blocked overnight with 5% non-fat milk in PBST buffer, followed by incubation with primary antibodies for 2 h: anti-GFP (Proteintech, Cat No. 66002–1-Ig) at a dilution of 3.5:10,000, or anti-mCherry (Proteintech, Cat No. 26765–1-AP) at a dilution of 3.5:10,000. The membrane was washed three times with PBST (fresh buffer replaced every 10 min), then incubated with an HRP-conjugated secondary antibody (Proteintech, Cat No. SA00001-1) for at least 45 min, and washed again three times with PBST (fresh buffer replaced every 10 min). Finally, the membrane was incubated with SuperSignal West Pico PLUS Chemiluminescent Substrate (ThermoFisher, Cat No. 34580) for 1 min, and images were captured using a Tanon 4600 Chemiluminescent Imaging System (Bio Tanon, China).

**Flg22-Triggered ROS Burst Assay**

*N. benthamiana* leaves transiently expressing the effector were harvested using a 5.5 mm diameter cork borer. The leaf discs were placed abaxially on water in a 96-well plate and incubated overnight in a growth chamber to allow for wound healing. The next day, the water was carefully aspirated to avoid damaging the leaves, and 200 μL of ROS assay solution (30 mg/L luminol, 20 mg/L horseradish peroxidase, 100 nM flg22 peptide) was added to each well. Chemiluminescence was immediately measured using a VICTOR Nivo™ Plate Reader.

**Flg22-Induced Callose Deposition Assay**

*N. benthamiana* leaves transiently expressing the effector were harvested using a 5.5 mm diameter cork borer. The leaf discs were immersed in 10 mM MgCl₂ containing 10 μM flg22 peptide for 24 h. The treated leaf discs were then transferred to 12-well plates containing 3 mL of decolorization solution (lactic acid: water-saturated phenol: glycerol: ethanol = 1:1:1:6) and incubated at 60°C with shaking (200 rpm) for 20 min. The decolorization solution was discarded, and the leaf discs were washed twice with 50% ethanol and once with distilled water. The leaf discs were then stained with 1 mL of aniline blue solution (150 mM K₂HPO₄, pH 9.5, 0.01% aniline blue) in the dark overnight. After staining, the leaf discs were washed gently with distilled water and observed under a fluorescence microscope (excitation: 365 nm; emission: 420 nm) to visualize callose deposition. Images were captured using a digital camera, and the fluorescence intensity was quantified using ImageJ software.

**Flg22-Induced Expression Analysis of Immune-Related Gene**

*N. benthamiana* leaves transiently expressing the effector were harvested using a 5.5 mm diameter cork borer at 12 h post-flg22 treatment. Total RNA was extracted from the leaf discs using the TransZol Up Plus RNA Kit. Genomic DNA contamination was removed using PrimeScript™ RT Reagent Kit with gDNA Eraser. First-strand cDNA was synthesized from 1 μg of total RNA and diluted 5-fold with nuclease-free water. Quantitative real-time PCR (qRT-PCR) was performed using the ChamQ Universal SYBR qPCR Master Mix. The reaction mixture (10 μL) contained 1 μL of diluted cDNA, 0.5 μL of each primer (*PR1a*-R: 5’-TCTCAACAGCCTTAGCAGCC-3’; *PR1a*-F: 5’-CGACCAGGTAGCAGCCTATG-3’; *PAL*-F: 5’-GTTATGCTCTTAGAACGTCGCCC-3’ and *PAL*-R: 5’-CCGTGTAATGCCTTGTTTCTTGA-3’; *Acre31*-F: 5’-AAGGTCCCGTCTTCGTCGGATCTTCG-3′; *Acre31*-R: 5′-AAGAATTCGGCCATCGTGATCTTGGTC-3′;), 3 μL of nuclease-free water, and 5 μL of 2× ChamQ Universal SYBR qPCR Master Mix. The reactions were run on a QuantStudio™ 5 Real-Time PCR System. The relative expression level of *PR1a* was calculated using the 2^(-ΔΔCt) method.

**Data Analysis**

The effector-target interaction network was visualized using Gephi (https://gephi.org/). Data visualization and statistical analysis were performed using GraphPad Prism 8.

**Reference**

González-Fuente, M., Carrère, S., Monachello, D., Marsella, B. G., Cazalé, A. C., Zischek, C.*, et al.* (2020) EffectorK, a comprehensive resource to mine for *Ralstonia*, *Xanthomonas*, and other published effector interactors in the *Arabidopsis* proteome. *Mol Plant Pathol,* **21,** 1257–1270.
