# Supplementary material for: Construction of an Effector–Target Interaction Network for Identification of Immune‐Related Effectors in Ralstonia pseudosolanacearum
Source: Mol Plant Pathol. 2026 Jun 9;27(6):e70280. doi: 10.1111/mpp.70280 (PMC13250401; doi:10.1111/mpp.70280)
Supplement: Supplementary file 5 — Table S1: Oligonucleotides and primers used in this study. [file MPP-27-e70280-s006.docx]

Table S1 Primer list used in this study.

| Primer name | Primer sequence (5'-3') |
| --- | --- |
| BD-RipO1F | tcagaggaggacctgATGCCAAAAATCCCAAAAAACC |
| BD-RipO1R | tcgacggatccccggTCAGGCGGCGGGGCTGGC |
| BD-RipS3F | tcagaggaggacctgATGGGCCTTGCACGACTGC |
| BD-RipS3R | tcgacggatccccggTTACCCCTCATAGCGATCGATC |
| BD-RipV1F | tcagaggaggacctgATGCCAACCCGCGTTCCG |
| BD-RipV1R | tcgacggatccccggCTAGCGGCTGCCTTGCGA |
| BD-RipAEF | tcagaggaggacctgTTGCTGAAAACGCGTATCACG |
| BD-RipAER | tcgacggatccccggTCAGTGCCTTTCCGGTGCC |
| BD-RipAOF | tcagaggaggacctgcatatgATGGCGGGTTCCCACTGC |
| BD-RipAOR | tcgacggatccccgggaattcTCAGCGGTACCCGTTCTGC |
| BD-RipP1F | tcagaggaggacctgATGAAAAGACTATTCAGAGCATTGGG |
| BD-RipP1R | tcgacggatccccggTCACGACTCCAGGGCATGTC |
| BD-RipG2F | tcagaggaggacctgATGGCCGCCCCGGTTTCC |
| BD-RipG2R | tcgacggatccccggCTACTCCGGAACGTCTTGCG |
| AD-UNE12-F | gccatggaggccagtgaattc ATGGCTAGTA ACAACCCTC |
| AD-UNE12-R | atgcccacccgggtggaattc CTACTGTGGAGGATTGTTCTC |
| AD-Kin7.4-F | gccatggaggccagtgaattc ATGGCTTCAT CCTCATCGAG |
| AD-Kin7.4-R | atgcccacccgggtggaattc TTAAGACGGAAACGCAAAGAGCCG |
| AD-KLCR2-F | gccatggaggccagtgaattc ATGGACGTAG GAGAGAGC |
| AD-KLCR2-R | atgcccacccgggtggaattc TCAATAAACCGGTCTCTGTCC |
| AD-OBE1-F | gccatggaggccagtgaattc ATGTTTTGGG CTTTCCTGAA TTAC |
| AD-OBE1-R | atgcccacccgggtggaattc CTAAGGATTGGATCTGAAAGG |
| AD-RCF3-F | gccatggaggccagtgaattc ATGGAGAGAT CTAGATCCAA G |
| AD-RCF3-R | atgcccacccgggtggaattc TCACGGTCCATCCTCTTGTATG |
| AD-AS1-F | gccatggaggccagtgaattc ATGAAAGAGA GACAACGTTG |
| AD-AS1-R | atgcccacccgggtggaattc TCAGGGGCGGTCTAATCTGC |
| AD-LIP5-F | gccatggaggccagtgaattc ATGTCGAACCCAAACGAACC |
| AD-LIP5-R | atgcccacccgggtggaattc TCAGTGACCGGCACCGGCCG |
| AD-TFPD-F | gccatggaggccagtgaattc ATGAATATCGTCTCTTGGAAAG |
| AD-TFPD-R | atgcccacccgggtggaattc TCACATATGGTGATCACTTCC |
| AD-APC8-F | gccatggaggccagtgaattc ATGGTCTCTAAAGAGTGTTG |
| AD-APC8-R | atgcccacccgggtggaattc CTAAATAGGAAAATGCTCG |
| AD-TIFY8-F | gccatggaggccagtgaattc ATGATGGTGAACCACAAC |
| AD-TIFY8-R | atgcccacccgggtggaattc TCATGTGGCTTCTTTTTCAGGATCTG |
| AD-F2N1-F | gccatggaggccagtgaattc ATGGTGACTCCGAAGCAGATC |
| AD-F2N1-R | atgcccacccgggtggaattc CTAACTATCATTAGCTGCC |
| mCherry-RipV1-F | CTGTACAAGGAGCTCGGTACC ATGCCAACCCGCGTTCCGTC |
| mCherry-RipV1-R | CTTGCATGCCTGCAGGTCGAC CTAGCGGCTGCCTTGCGAAG |
| mCherry-RipO1-F | CTGTACAAGGAGCTCGGTACC ATGCCAAAAATCCCAAAAAAC |
| mCherry-RipO1-R | CTTGCATGCCTGCAGGTCGAC TCAGGCGGCGGGGCTGGCCG |
| mCHerry-RipAE-F | CTGTACAAGGAGCTCGGTACC TTGCTGAAAACGCGTATCACG |
| mCHerry-RipAE-R | CTTGCATGCCTGCAGGTCGAC TCAGTGCCTTTCCGGTGCC |
| mCHerry-RipAO-F | CTGTACAAGGAGCTCGGTACC ATGGCGGGTTCCCACTGC |
| mCHerry-RipAO-R | CTTGCATGCCTGCAGGTCGAC TCAGCGGTACCCGTTCTGC |
| mCHerry-RipS3-F | CTGTACAAGGAGCTCGGTACC ATGGGCCTTGCACGACTGC |
| mCHerry-RipS3-R | CTTGCATGCCTGCAGGTCGAC TTACCCCTCATAGCGATCGATC |
| mCHerry-RipG2-F | CTGTACAAGGAGCTCGGTACC ATGGCCGCCCCGGTTTCC |
| mCHerry-RipG2-R | CTTGCATGCCTGCAGGTCGAC CTACTCCGGAACGTCTTGCG |
| RipV1-cYFP-F | GGCGCGCCACTAGTGGATCC ATGCCAACCCGCGTTCCGTC |
| RipV1-cYFP-R | TCGTATGGGTACATCCCGGG CTAGCGGCTGCCTTGCGAAG |
| nYFP-RCF3-R | GGCGCGCCACTAGTGGATCC ATGGAGAGATCTAGATCCAAG |
| nYFP-RCF3-R | AACTTTTGCTCCATCCCGGG TCACGGTCCATCCTCTTGTATG |
| nYFP-OBE1-R | GGCGCGCCACTAGTGGATCC ATGTTTTGGGCTTTCCTGAATTAC |
| nYFP-OBE1-R | AACTTTTGCTCCATCCCGGG CTAAGGATTGGATCTGAAAGG |
| nYFP-KLCR2-R | GGCGCGCCACTAGTGGATCC ATGGACGTAGGAGAGAGC |
| nYFP-KLCR2-R | AACTTTTGCTCCATCCCGGG TCAATAAACCGGTCTCTGTCC |
| \| QRT-OBE1-F \| \| --- \| \| QRT-OBE1-R \| \| QRT-F2N1-F \| \| QRT-F2N1-R \| \| QRT-APC8-F \| \| QRT-APC8-R \| \| QRT-KLCR2-F \| \| QRT-KLCR2-R \| \| AtACT2-F \| \| AtACT2-R \| | \| CATCTGGGTCCAATCTTC \| \| --- \| \| GTCTTTCCGCTATCTGTC \| \| GGCGAGAAACTCACGACAC \| \| CCACCAGCAAGAGGATGAC \| \| TCAACACCGTTACCTTCC \| \| TCTCCAGCCAGATAAAGC \| \| AATGACGCTGTGCCTGTTC \| \| CAACTCGTGCATCCGATTCTC \| \| CGCTCTTTCTTTCCAAGCTCAT \| \| TGCATCCTTCTGGTTCATCCC \| |
